# Microbiology of parapharyngeal abscesses in adults: in search of the significant pathogens

**DOI:** 10.1007/s10096-021-04180-y

**Published:** 2021-02-10

**Authors:** Tejs Ehlers Klug, Thomas Greve, Camilla Andersen, Pernille Hahn, Christian Danstrup, Niels Krintel Petersen, Mirjana Ninn-Pedersen, Sophie Mikkelsen, Søren Pauli, Simon Fuglsang, Helle Døssing, Anne-Louise Christensen, Maria Rusan, Anette Kjeldsen

**Affiliations:** 1 Department of Otorhinolaryngology, Head & Neck Surgery, Aarhus University Hospital, Palle Juul-Jensens Boulevard 99, DK-8200 Aarhus N, Denmark; 2 Department of Clinical Microbiology, Aarhus University Hospital, Aarhus, Denmark; 3 Department of Otorhinolaryngology, Head & Neck Surgery, Hospital Lillebaelt, Kolding, Denmark; 4 Department of Otorhinolaryngology, Head & Neck Surgery, Aalborg University Hospital, Aalborg, Denmark; 5 Department of Otorhinolaryngology, Head & Neck Surgery, Odense University Hospital, Odense, Denmark; 6 Department of Otorhinolaryngology, Head & Neck Surgery, Regional Hospital West Jutland, Herning, Denmark

**Keywords:** Parapharyngeal abscess, Microbiology, *Fusobacterium*, *Streptococcus*, Pathogens

## Abstract

We aimed to describe the microbiology of parapharyngeal abscess (PPA) and point out the likely pathogens using the following principles to suggest pathogenic significance: (1) frequent recovery, (2) abundant growth, (3) growth in relative abundance to other microorganisms, (4) percentage of the isolates recovered in both absolute and relative abundance, (5) more frequent recovery in PPA pus compared with tonsillar surface and tissue. Comprehensive bacterial cultures were performed on specimens obtained from adult patients (*n* = 60) with surgically verified PPA, who were prospectively enrolled at five Danish ear-nose-throat departments. The prevalent isolates (in PPA pus) were unspecified anaerobes (73%), non-hemolytic streptococci (67%), *Streptococcus anginosus* group (SAG) (40%), *Corynebacterium* spp. (25%), *Neisseria* spp. (23%), *Fusobacterium* spp. (22%), *Fusobacterium necrophorum* (17%), *Prevotella* spp. (12%), and *Streptococcus pyogenes* (10%). The bacteria most frequently isolated in heavy (maximum) growth were unspecified anaerobes (60%), SAG (40%), *F. necrophorum* (23%), and *Prevotella* spp. (17%). The predominant microorganisms (those found in highest relative abundance) were unspecified anaerobes (53%), SAG (28%), non-hemolytic streptococci (25%), *F. necrophorum* (15%), *S. pyogenes* (10%), and *Prevotella* spp. (10%). Four potential pathogens were found in both heavy growth and highest relative abundance in at least 50% of cases: *F. necrophorum*, *Prevotella* spp., SAG, and *S. pyogenes*. SAG, *Prevotella* spp., *F. necrophorum*, *S. pyogenes*, and *Bacteroides* spp. were recovered with the same or higher frequency from PPA pus compared with tonsillar tissue and surface. Our findings suggest that SAG, *F. necrophorum*, Prevotella, and *S. pyogenes* are significant pathogens in PPA development.

## Introduction

Parapharyngeal abscess (PPA) refers to a collection of pus located laterally or posteriorly to the pharyngeal constrictor muscle. The pathogenesis of PPA is scarcely described but likely includes lymphogenous or direct spread of bacteria from upper airway mucosa or teeth [1]. Less frequently, PPAs are reported as extensions of peritonsillar abscesses (PTA) [2–5].

As with other neck abscesses derived from upper airway mucosa (i.e., PTA), a polymicrobial mixture of aerobes and anaerobes can be grown from PPA pus, when appropriate culture methods are applied [6, 7]. These polymicrobial infections derived from areas that are heavily colonized raise the following questions: which bacteria are significant pathogens, which are merely non-pathogenic bystanders, and which bacteria represent contamination as the needle pass through the mucosa and tissues to the abscess?

The knowledge regarding significant pathogens associated with PPA is very limited. The few previous studies focusing on the microbiology of PPA in adults were all retrospective, and thus reported findings in routine cultures, and no attempts were made to analyze the significance of the recovered bacteria [2, 5, 8, 9].

The current study was undertaken to further describe the microbiology associated with PPA in adults, using comprehensive aerobic and anaerobic culture methods, and to identify which bacteria were the likely pathogens.

## Materials and methods

### Patients

Patients were prospectively enrolled in the study between April 2016 and August 2019 at five Danish ear-nose-throat departments (Aarhus University Hospital, Odense University Hospital, Aalborg University Hospital, Hospital Lillebaelt, and Regional Hospital West Jutland). The inclusion criteria were (1) patients with PPA, defined as surgical finding of pus located laterally or posteriorly to the pharyngeal constrictor muscle, including abscesses in the parapharyngeal, retropharyngeal, visceral, and pharyngeal mucosa (the part laterally to the constrictor muscle) spaces, but excluding base of tongue, floor of mouth, and submandibular space, (2) age > 17 years, and (3) written and oral consent.

Patients were categorized in subgroups based on the site of primary infection and the presence of concurrent PTA.

### Specimen collection

After the induction of general anaestesia, swabs were rubbed thoroughly on the surfaces of each of the tonsils and placed in transport media (E-swab (Copan, Brescia, Italy)). The PPA was punctured through the pharyngeal mucosa or externally through the skin and aspirated into a sterile syringe. In cases of concurrent PTA, needle aspiration was performed through the peritonsillar mucosa to the PTA. If pus was not obtained through intact mucosa or skin, pharyngeal (+/− ipsilateral tonsillectomy) or skin incision was performed, and pus was collected into a sterile syringe at the time of abscess perforation. In cases of tonsillectomy, large bilateral tonsil biopsies (approximately half the volume of the tonsils) were obtained and placed in sterile containers separately. Pus aspirates, tonsillar tissue, and surface swabs were placed at −80 ^o^C within 30 min of collection.

### Microbiological analyses

Samples were processed in a class 2 laminar air flow safety cabinet using an aseptic technique. Initially, a pilot study was performed to determine which culture media were optimal for culturing in relation to bacterial distinction. In ten patients, pus aspirates, tissue samples, and swabs were plated on 5% horse blood agar, chocolate ager, 10% horse blood agar, anaerobic agar (chocolate plate containing K-vitamin and cysteine), selective *Fusobacterium* agar (containing 5 mg/L nalidixic acid and 2.5 mg/L vancomycin) [10], and semi-solid thioglycolate (Statens Serum Institute Diagnostica, Hillerød, Denmark). Plates were incubated at 35 °C, the first three plates in 5% CO_2_, the next two plates in anaerobic atmosphere including a metronidazole-disc (10 UG) (Oxoid, Roskilde, Denmark), and the thioglycolate vial at ambient atmosphere. A Mueller-Hinton agar with horse blood and 20 mg/L NAD (Oxiod, Roskilde, Denmark), plus selected antimicrobial discs, was also inoculated and incubated at 35 °C at 5% CO_2_; this plate was not used for antimicrobial susceptibility testing but as a selective medium aiding the initial differentiation of bacterial species. All plates were incubated for up to 7 days and checked for growth after 2 and 4 days. Based on the pilot study, the rest of the samples were plated on 5% horse blood agar, anaerobic agar, *Fusobacterium* agar, Mueller-Hinton agar and thioglycolate, and checked for growth after 2 and 4 days. The thioglycolate medium was only cultured when the solid media showed no growth after 4 days of incubation. Cultured microorganisms were identified by phenotypic appearance and biochemical profiles according to accepted guidelines [11] and in selected cases supplemented with matrix-assisted laser desorption/ionization time-of-flight mass spectrometry (MALDI-TOF MS) (Bruker Daltonics, Bremen, Germany). The microorganism colony count was reported semi-quantitatively.

Species level identification was performed depending on the type of material (pus, tissue, or swab), abundance of the bacteria, and methods available for identification. In general, bacteria belonging to the normal pharyngeal flora were described at genus level in tissue and swab cultures (e.g., non-hemolytic streptococci, *Neisseria* spp.*, Corynebacterium* spp.), whereas bacteria were identified to species level in pus if they were in a dominant or co-dominant quantity. Species differentiation between the closely related *Streptococcus anginosus*, *Streptococcus constellatus,* and *Streptococcus intermedius* is inadequate by phenotypic appearance, MALDI-TOF, and 16S rDNA gene fragment analysis and is thus referred to as *Streptococcus anginosus* group. Most anaerobic bacteria were merged into unspecified anaerobes, where the initial criteria were growth in anaerobic atmosphere and sensitivity towards metronidazole. Long wave UV light was used to detect characteristic fluorescence of the colonies (e.g., *Fusobacterium* spp. = green, *Prevotella* spp., *Porphyromonas* spp., and *Veillonella* spp*.* = red), and pigmentation was also used for identification (e.g., *Prevotella* spp. = black) [12]. *Fusobacterium* was divided into *Fusobacterium nechrophorum* and *Fusobacterium* spp. (the latter including as per MALDI-TOF identification: *Fusobacterium nucleatum*, *Fusobacterium naviforme*, *Fusobacterium gondiaformans*, and *Fusobacterium sp*.). All species of Prevotella are referred to as *Prevotella* spp. (containing as per MALDI-TOF identification: *Prevotella oris, Prevotella denticola, Prevotella disiens, Prevotella nigrescens, Prevotella baroniae*, and *Prevotella histocola*), Veillonella as *Veillonella* spp., and Bacteroides is divided into *Bacteroides fragilis* and *Bacteroides* spp.

### Pathogenic significance

To identify likely pathogens, we considered several factors, including absolute and relative abundance of growth in cultures from PPA pus, as well as recovery from PPA pus vs. the tonsillar mucosa/tissue. In this regard, we assumed that frequent recovery of a microorganism in absolute and relative (compared with the other bacteria) abundance at the site of infection (PPA pus) suggests pathogenic significance [13]. To address the problem with insignificant bystanders and contamination, we hypothesized that more frequent recovery from PPA pus than from the tonsillar mucosa points to pathogenic significance.

Hence, the following principles to suggest pathogenic significance of the recovered bacteria were used in the current study: (1) frequent (10% or more) recovery in PPA pus, (2) abundant (maximal level) growth in PPA pus, (3) greater relative abundance in PPA pus culture compared with other microorganisms, (4) majority (50% or more) of isolates recovered in both absolute and relative abundance, (5) more frequent recovery in PPA pus compared with tonsillar surface swabs and tonsillar tissue.

### Statistical analyses

Statistical analyses were performed using the Fisher’s exact test for categorical variables (absolute number of isolates) and the Student *t*-test and analysis of variance (ANOVA) for continuous variables (mean number of isolates and biochemical parameters) in between-groups comparisons. The binomial probability test was used for gender comparison. The normality of data was assessed using quantile-quantile (QQ) plots. Statistical significance was defined as *p* < 0.05.

### Permissions

The study was approved by the Ethical Committee of Aarhus County (# 1-10-72-4-16) and by the Danish Data Protection Agency (1-16-02-65-16). Informed consent was obtained from all patients, in accordance with the guidelines set by the Danish National Board of Health.

## Results

### Patient characteristics

Sixty adult patients (mean age 51 years, range 18–89 years) with surgically verified PPA were enrolled in the study. Twenty-six (43%) patients had concurrent PTA. Significantly more patients were male (*n* = 43, 72%) (*p* = 0.001, binomial probability test). The oropharynx was deemed (by the investigator) as the primary site of infection in the majority of cases (*n* = 48, 80%), followed by the hypopharynx (*n* = 8, 13%), the larynx (*n* = 2, 3%), and the teeth (*n* = 1, 2%). One patient had unknown site of primary infection. Six (10%) patients were previously tonsillectomized (all bilaterally). The majority of patients had sore throat (98%), pain on swallowing (97%), and anamnestic fever (67%). Clinical findings included parapharyngeal swelling (97%, on flexible endoscopy), peritonsillar (71%) and tonsillar swelling (63%), tender neck on palpation (67%), trismus (58%), neck swelling (43%), dyspnea (25%), and torticollis (10%). Antibiotics were prescribed to 51% (30/59) of patients prior to admission, and 86% (48/56) received antibiotics before specimens were obtained (Table 1). No statistically significant differences in biochemical parameters were found between subgroups (based on the site of primary infection and the presence of concurrent PTA; Table 1).

**Table 1:** Clinical and biochemical characteristics of 60 patients with parapharyngeal abscess stratified by primary site of infection and presence of concurrent peritonsillar abscess (PTA)

Primary site of infection	All *n* = 60	Oropharynx *n* = 48		Hypopharynx *n* = 8	Other^1^ *n* = 4
		+ PTA *n*=26	- PTA *n*=22		
Males	43 (72%)	18 (69%)	17 (77%)	5 (63%)	3 (75%)
Age, mean (SD)^2^	51.3 (19)	47.3 (21)	54.2 (16)	48.3 (17)	67.0 (10)
Duration of symptoms, median (days)	4.5	5.0	4.0	4.0	4.5
Antibiotic treatment prior to admission^3^	51%	60%	40%	63%	25%
Penicillin V	40%	48%	40%	38%	0%
Penicillin V + metronidazole	9%	12%	0%	25%	0%
Amoxicillin-clavulanate	2%	0%	0%	0%	25%
Antibiotic treatment prior to obtaining specimens^4^	86%	87%	82%	88%	75%
Tobacco smoking (current)^5^	41%	46%	38%	25%	50%
Temperature, mean °C (SD)^6^	38.0 (0.8)	38.0 (0.7)	37.9 (0.8)	38.1 (1.0)	39.3
Biochemistry, mean (SD)					
C-reactive protein (mg/L)^7^	186 (106)	177 (111)	176 (104)	192 (55)	299 (141)
Leukocyte count (× 10^9^/L)^8^	15.4 (3.6)	14.6 (2.8)	15.1 (4.2)	17.2 (3.9)	18.3 (1.8)
Neutrophil count (× 10^9^/L)^9^	12.4 (3.2)	11.9 (2.5)	11.8 (3.5)	14.3 (4.0)	15.7 (2.5)

^1^The larynx (*n* = 2), tooth (*n* = 1), unknown (*n* = 1)

^2^Subgroup comparison: *p* = 0.19, ANOVA

^3^Three patients with missing information (*n* = 57)

^4^Pencillin (*n* = 17), penicillin and metronidazole (*n* = 14), cefuroxime and metronidazole (*n* = 10), cefuroxime (*n* = 5), and ampicillin (*n* = 2)

^5^*n* = 59

^6^*n* = 36

^7^Subgroup comparison: *p* = 0.18, ANOVA), *n* = 59

^8^Subgroup comparison: *p* = 0.15, ANOVA), *n* = 59

^9^Subgroup comparison: *p* = 0.07, ANOVA), *n*=55

### Number of isolates

The mean number of isolates was significantly higher in PTA pus (4.3, SD 1.8) than PPA pus (3.4, SD 1.5) (*p* = 0.038, Student’s *t*-test) and higher in ipsilateral tonsillar tissue (5.7, SD 1.6) and surface swabs (5.8, SD 1.6) (both *p* < 0.001, Student’s *t*-test). No significant differences were found between the mean numbers of isolates in cultures from ipsi- and contralateral tonsillar tissues (*p* = 0.65, Student’s *t*-test) or from ipsi- and contralateral surface swabs (*p* = 0.95, Student’s *t*-test).

The mean number of isolates from PPA and PTA pus cultures was similar between the pilot study (samples from ten patients cultured on a larger variety of plates) compared with the rest of the samples (PPA 3.8 vs 3.3, *p* = 0.37, Student’s *t*-test; PTA 4.7 vs 4.3, *p* = 0.73).

### PPA pus culture findings

PPA pus cultures were polymicrobial in 93% (56/60) of cases. The most prevalent isolates were unspecified anaerobes (*n* = 44, 73%), non-hemolytic streptococci (*n* = 40, 67%), *Streptococcus anginosus* group (*n* = 24, 40%), *Corynebacterium* spp. (*n* = 15, 25%), *Neisseria* spp. (*n* = 14, 23%), *Fusobacterium* spp. (*n* = 13, 22%), *F. necrophorum* (*n* = 10, 17%), *Prevotella* spp. (*n* = 7, 12%), and *Streptococcus pyogenes* (*n* = 6, 10%) (Table 2). No statistically significant differences in recovery rates were found between subgroups (Table 2). Furthermore, no significant impact of smoking, age (cut off 50 years), or the presence of PTA was found on culture findings.

**Table 2:** Culture findings in parapharyngeal pus aspirates among 60 patients with parapharyngeal abscess stratified by primary site of infection and presence of concurrent peritonsillar abscess (PTA)

Primary site of infection	All *n* = 60	Oropharynx *n* = 48		Hypopharynx *n* = 8	Other^1^ *n* = 4
Microorganisms		+PTA *n* = 26	−PTA *n* = 22		
Aerobic bacteria					
*Streptococcus pyogenes*	6 (10%)	1 (4%)	4 (18%)		1 (25%)
*Streptococcus anginosus* group	24 (40%)	12 (46%)	10 (45%)	1 (13%)	1 (25%)
Non-hemolytic streptococci	40 (67%)	17 (65%)	14 (64%)	6 (75%)	3 (75%)
*Streptococcus pneumoniae*	2 (3%)		2 (9%)		
*Haemophilus parainfluenzae*	1 (2%)		1 (5%)		
*Staphylococcus aureus*	2 (3%)	1 (4%)	1 (5%)		
*Coagulase-negative staphylococci*	4 (7%)	2 (8%)	1 (5%)	1 (13%)	
*Eikenella corrodens*	4 (7%)	2 (8%)	1 (5%)	1 (13%)	
*Neisseria* spp.	14 (23%)	2 (8%)	7 (32%)	5 (63%)	
*Escherichia coli*	2 (3%)	2 (8%)			
*Proteus vulgaris*	1 (2%)	1 (4%)			
*Aggregatibacter aphrophilus*	1 (2%)		1 (5%)		
*Corynebacterium* spp.	15 (25%)	6 (23%)	6 (27%)	2 (25%)	1 (25%)
*Trueperella pyogenes*	1 (2%)		1 (5%)		
*Gemella* spp.	1 (2%)			1 (13%)	
Anaerobic bacteria					
*Fusobacterium necrophorum*	10 (17%)	5 (19%)	3 (14%)	2 (25%)	
*Fusobacterium* spp.	13 (22%)	4 (15%)	7 (32%)		2 (50%)
*Prevotella* spp.	7 (23%)	5 (19%)	2 (1%)		
*Bacteroides fragilis*	1 (2%)		1 (5%)		
*Bacteroides spp.*	1 (2%)	1 (4%)			
*Bifidobacterium longum*	1 (2%)	1 (4%)			
*Eggerthia catenaformis*	1 (2%)		1 (5%)		
*Capnocytophaga* spp.	1 (2%)	1 (4%)			
*Actinomyces* spp.	2 (3%)			1 (13%)	1 (25%)
*Alloscardovia omnicolens*	1 (2%)	1 (4%)			
*Lachnoanaerobaculum orale*	1 (2%)			1 (13%)	
Anaerobes (unspecified)	44 (73%)	17 (65%)	18 (82%)	7 (88%)	2 (50%)
Fungi					
Candida	1 (2%)	1 (4%)			
Number of isolates, mean	3.4	3.2	3.7	3.5	2.8
Polymicrobial	56 (93%)	22 (85%)	22 (100%)	8 (100%)	4 (100%)

^1^The larynx (*n* = 2), tooth (*n* = 1), unknown (*n* = 1)

### Abundant growth

Among the 35 PPA patients who had heavy growth (the maximal level of growth) of one or more microorganisms, the bacteria most frequently recovered in heavy growth in PPA pus specimens were unspecified anaerobes (*n* = 21, 60%), *Streptococcus anginosus* group (*n* = 14, 40%), *F. necrophorum* (*n* = 8, 23%), and *Prevotella* spp. (*n* = 6, 17%) (Table 3).

**Table 3:** Predominant microbiologic findings in parapharyngeal abscess (PPA) pus based on semi-quantitative cultures

Microorganisms	Relative abundance^1^ *n* = 60	Absolute abundance^2^ *n* = 35	Percentage of isolates found in relative/absolute abundance^3^
Aerobic bacteria			
*Streptococcus pyogenes*	6	3	100%/50%
*Streptococcus anginosus* group	17	14	71%/58%
Non-hemolytic streptococci	15	6	38%/15%
*Streptococcus pneumoniae*	1		50%/0%
*Staphylococcus epidermidis*	1		25%/0%
*Eikenella corrodens*	2		50%/0%
*Aggregatibacter aphrophilus*			0%/0%
*Neisseria* spp.	2		14%/14%
*Escherichia coli*			0%/0%
*Corynebacterium* spp.	2		13%/0%
*Gemella* spp.			0%/0%
Anaerobic bacteria			
*Fusobacterium necrophorum*	9	8	90%/80%
*Fusobacterium* spp.	5	4	38%/31%
*Prevotella* spp.	6	6	86%/86%
*Bacteroides* spp.	1	1	50%/50%
*Bifidobacterium longum*	1		100%/0%
*Eggerthia catenaformis*	1	1	100%/100%
Capnocytophaga spp.	1	1	100%/100%
*Actinomyces* spp.	1	1	100%/100%
*Lachnoanaerobaculum orale*	1	1	100%/100%
Anaerobes (unspecified)	32	21	73%/48%
Fungi			
Candida	1		50%/0%

^1^Number (%) of microorganisms isolated in maximum (either heavy (*n* = 35), moderate (*n* = 19), or sparse (*n* = 6)) semi-quantitative growth among patients with PPA

^2^Number (%) of microorganisms isolated in heavy semi-quantitative growth among patients with PPA

^3^See Table 2 for total number of microorganisms

### Growth in relative abundance to other microorganisms

The most frequently recovered predominant microorganisms (most abundant in relation to other isolates in the same culture) in PPA pus specimens were unspecified anaerobes (*n* = 32, 53%), *Streptococcus anginosus* group (*n* = 17, 28%), non-hemolytic streptococci (*n* = 15, 25%), *F. necrophorum* (*n* = 9, 15%), *S. pyogenes* (*n* = 6, 10%), and *Prevotella* spp. (*n* = 6, 10%) (Table 3).

### Majority of isolates recovered in both heavy growth and relative abundance

Four potential pathogens were found in both heavy growth and highest relative abundance in at least 50% of cases: *F. necrophorum* (90% of isolates were found in heavy growth/80% of isolates were found in highest relative abundance), *Prevotella* spp. (86%/86%), *Streptococcus anginosus* group (71%/58%), and *S. pyogenes* (100%/50%) (Table 3). In addition, a number of infrequent recoveries were also found in both relative and absolute abundance (*Bacteroides sp.* (*n* = 1), *Eggerthia catenaformis* (*n* = 1), *Capnocytophaga sp.* (*n* = 1), and *Lachnoanaerobaculum orale* (*n* = 1)) (Table 3).

### PPA pus versus tonsillar tissue and surface swab findings

In 53 patients, a complete set of ipsilateral cultures was performed (including PPA pus, ipsilateral tonsillar tissue, and ipsilateral tonsillar swab). Disregarding isolates detected in only one patient, *Streptococcus anginosus* group, *Prevotella* spp., *F. necrophorum*, *S. pyogenes*, and *Bacteroides* spp. were recovered with the same or higher frequency from PPA pus compared with tonsillar tissue and tonsillar surface swabs (Table 4). In contrast, *Corynebacterium* spp., *Neisseria* spp., and *Hemophilus* spp. were recovered from PPA pus much less frequently compared with tonsillar tissue and swab (Table 4).

**Table 4:** Bacterial findings in cultures from parapharyngeal abscess (PPA) pus, tonsillar tissue (ipsilateral to PPA), and tonsillar surface swab (ipsilateral to PPA) among 53 PPA patients with all three specimens collected

	PPA pus	Tonsillar tissue	Tonsillar surface swab
Aerobic bacteria			
*Streptococcus pyogenes*	5	4	5
*Streptococcus anginosus* group	21	20	8
Non-hemolytic streptococci	36	50	51
*Streptococcus pneumoniae*	2	1	1
*Streptococcus agalactiae*		2	2
*Haemophilus influenzae*		3	1
*Haemophilus parainfluenzae*	1	4	10
*Haemophilus haemolyticus*			1
*Haemophilus parahaemolyticus*			1
*Staphylococcus aureus*	2	7	9
*Staphylococcus lugdunensis*			1
*Coagulase-negative staphylococci*	3	25	31
*Eikenella corrodens*	3	8	10
*Aggregatibacter aphrophilus*	1		
*Neisseria* spp.	12	38	34
*Escherichia coli*	2	3	3
*Proteus vulgaris*	1		
*Corynebacterium* spp.	13	39	38
*Mycoplasma* sp.^1^		1	1
*Gemella* sp.	1	1	
Anaerobic bacteria			
*Fusobacterium necrophorum*	8	8	7
*Fusobacterium* spp.	13	18	8
*Prevotella* spp.	8	5	5
*Bacteroides* spp.	2^2^	2^3^	3^4^
*Bifidobacterium longum*	1		
*Eggerthia catenaformis*	1		
*Capnocytophaga* spp.	1	1	2
*Actinomyces* spp.	1	1	1
*Veillonella* spp.		8	7
*Lactobacillus* spp.		2	3
*Parvimonas micra*	1		
*Lachnoanaerobaculum orale*	1		
*Alloscardovia omnicolens*	1		
Anaerobes (unspecified)	37	49	48
Fungi			
Candida	2	4	14
No. isolates, mean (SD)	3.4 (1.5)	5.8 (1.6)	5.9 (1.6)
Polymicrobial	50 (94%)	52 (98%)	53 (100%)

^1^*M. hominis* (*n* = 1)

^2^*B. fragilis* (*n* = 1), *B. pyogenes* (*n* = 1)

^3^*B. fragilis* (*n* = 2)

^4^*B. fragilis* (*n* = 3)

### The impact of antibiotic treatment prior to admission

The mean number of isolates in PPA cultures among patients without antibiotic treatment prior to admission was higher (3.7) compared with patient with antibiotic treatment (3.1), although this was not statistically significant (*p* = 0.16, Student’s *t*-test). Similarly, polymicrobial growth was found in all PPA pus cultures from patients without antibiotic treatment before admission compared with 86% in patients, who had received antibiotics (*p* = 0.11, Fisher’s exact test). *S. pyogenes* was recovered significantly more frequently among patients without antibiotic treatment prior to admission (21%) compared with patients treated with antibiotics (0%) (*p* = 0.01, Fisher’s exact test).

## Discussion

### Suggested significant pathogens

Based on extensive cultures from PPA aspirates obtained with a focus to minimize the contamination from surrounding tissues, a polymicrobial aerobic and anaerobic flora was found in the vast majority (93%) of PPAs, and the most frequently recovered bacteria were non-hemolytic streptococci (including the *Streptococcus anginosus* group) and unspecified anaerobes.

Focusing on the absolute and relative abundance of the obtained microorganisms, unspecified anaerobes, *Streptococcus anginosus* group, *Prevotella* spp., and *F. necrophorum* stand out as the prevalent recoveries. To further pinpoint the likely pathogens and deduct the massive commensal flora of the pharynx, we (1) calculated the percentage of each bacterial strain, which was most commonly recovered in both absolute and relative abundance and (2) compared the findings in PPA pus with tonsillar tissues and surface swabs. These analyses suggested that the *Streptococcus anginosus* group, *F. necrophorum*, *Prevotella* spp., and *S. pyogenes* were the major pathogens. Fifty-three percent (32/60) of patients had one or more of these four bacterial strains recovered in absolute or relative abundance from their PPA, and one or more of the bacteria were obtained in 67% (40/60) of patients (regardless of abundance). The less frequent detection of *Streptococcus anginosus* group from tonsillar swabs compared with PPA pus and tonsillar tissue might suggest that *Streptococcus anginosus* group is part of the commensal flora of the tonsillar crypts and therefore not sampled adequately by tonsillar swaps. Alternatively, these findings could support the pathogenic role of *Streptococcus anginosus* group in the anaerobe micro-conditions of PPA. Unspecified anaerobes and non-hemolytic streptococci (other than the *Streptococcus anginosus* group) were also frequently recovered, and further investigations are warranted to explore the potential roles for subgroups of bacteria within these categories. *S. pyogenes* was recovered significantly less frequently in patients who were treated with antibiotics prior to admission (0%) compared with untreated patients (21%), which may suggest that this pathogen is underestimated in our findings.

### Previous studies of PPA microbiology

Itzhak Brook performed extensive cultures on PPA pus specimens from 14 children aged 1–6 years in the only previous prospective microbiologic study of PPA patients [6]. A polymicrobial mixture of aerobes and anaerobes was found in 12 cases, and only anaerobes were detected in the latter two cases. The predominant bacteria were *Bacteroides* spp. (*n* = 32), *Peptostreptococcus* spp. (*n* = 18), alpha- and gamma-hemolytic streptococci (*n* = 14), *F. nucleatum* (*n* = 6), *Staphylococcus aureus* (*n* = 5), and *Fusobacterium* spp. (*n* = 5). Retrospective, pediatric studies from the same period (late 80s) reported similar findings [14, 15]. It is well described in other pharyngeal infections (i.e., acute tonsillitis and PTA) that the prevalent pathogens are closely associated with patient age, and it is doubtful that these pediatric findings can be extrapolated to adults [7, 16].

Only a few studies of adult PPA patients include information on the microbiology, and no previous attempts to exhaustively define the bacteriology have been done [2, 5, 8, 9, 17]. Hence, previous studies were retrospective and described routine culture findings. Sethi et al. reported the recovery of four *Klebsiella pneumoniae*, one *Pseudomonas aeruginosa*, and one *S. aureus* among nine patients [9]. Alaani et al. described five PPA cases with different combinations of streptococci (beta-hemolytic, milleri group, and unspecified) and anaerobes (*Peptostreptococcus* spp., *Bacteroides* (currently named *Prevotella*) *melaninogenicus*, and unspecified) [8]. Among three patients with PTA and PPA, Ohori et al. reported two ahd positive cultures for *S. constellatus*, alone and in combination with *P. melaninogenica* in each case. Thapar et al. found *S. pyogenes* and mixed anaerobes in blood cultures from a 24-year-old woman with PPA [17]. Lastly, in an earlier retrospective study from our own group of 61 PPA patients, 28 patients had positive culture findings from pus aspirate or pus swabs, and the predominant bacterial species were *S. pyogenes*, viridans group streptococci, *F. necrophorum*, and unspecified anaerobes [5]. Hence, a wide variety of bacterial species has been grown from PPA pus specimens, but the very limited previous evidence points in the same direction as the findings in the current study. It is noteworthy that 35 of 79 (44%) PPA patients in these previous studies had concomitant PTA, which is in accordance with the current study (43%), and that the recovered bacterial flora is comparable for those with or without concomitant PTA.

### Previous evidence to suggest pathogenic importance of individual bacteria in throat infections

The significance of *S. pyogenes*, *F. necrophorum*, *Prevotella* spp., and the *Streptococcus anginosus* group in PPA has only had limited attention in previous studies. However, it seems reasonable to extrapolate from other throat infections to PPA.

*S. pyogenes* is widely recognized as the major pathogen in throat infections. In acute tonsillitis, it has been recovered more frequently among patients than healthy controls, the development of specific antibodies has been documented, and antibiotics reduce the duration of symptoms and the risk of PTA and acute rheumatic fever [18–22]. In PTA, *S. pyogenes* has been consistently detected in pus aspirate studies, the development of specific antibodies has been documented, and it has been recovered more frequently in tonsillar core tissue from PTA patients than controls [7, 23–25].

*F. necrophorum* is a well-characterized pathogen known to cause Lemierre’s syndrome [26]. The role for *F. necrophorum* in acute tonsillitis is unclarified, but a number of studies report more frequent detection among patients than healthy controls [27]. Numerous findings suggest that it is a significant pathogen in PTA, given high recovery rates in PTA pus aspirates, more frequent isolation from PTA patients compared with controls, significantly higher inflammatory markers in *F. necrophorum*–positive patients compared with patients infected with other bacteria, and the development of specific antibodies [7, 25, 28]. In the current study, *F. necrophorum* was found in 17% of PPA pus specimens, which is considerably less than the previous studies of PTA, where anaerobic cultures were performed (38–58%) [7, 29]. This less prevalent role of *F. necrophorum* may be related to the fact that this anaerobe has a high preponderance among patients aged 15–30 years, which coincides with the highest incidences of Lemierre’s syndrome, PTA, and acute tonsillitis but is considerably younger than the majority of PPA patients [26, 27, 30]. In that regard, it is noteworthy that *F. necrophorum* also seems to play a role in PPA and that the mean age of *F. necrophorum*–positive patients was 48 years in the current study and not significantly different from patients with other bacterial findings.

The significance of *Prevotella* spp. in throat infections is uncertain. In the 1990s, Brook and colleagues performed a number of serological studies and reported an increase (at least a doubling) in antibody levels to *Prevotella intermedia* in patients with PTA, peritonsillar cellulitis, infectious mononucleosis, acute tonsillitis, and recurrent non-streptococcal tonsillitis [31–34].

The *Streptococcus anginosus* group is known to cause a variety of different human infections, but the pathogenic importance in throat infections is previously undocumented [35].

### Limitations

The current study has several limitations. The relatively limited number of patients reflects the relative low incidence of PPA. Our findings are limited to adults, and the pathogens associated with PPA in children are likely different. Due to the large number of specimens and to homogenize processing, specimens were kept at −80 °C until cultures were made. Although studies on the effect of freezing specimens do not seem to alter the ability to isolate microorganisms, some bacteria may not have been detected, i.e., fastidious bacteria or bacteria, which had been killed before the collection of specimens (most patients were treated with antibiotics). This study relies on classical bacteriology where a correct and clinically relevant species distinction depends on the skills of the clinical laboratory technician. Initial identification of organisms is grounded in subjective criteria supplied with biochemical reactions, and experience is established through the years [36]. To minimize this processing bias, the same very experienced technician processed all samples in the current study. The use of selective and differential media for initial processing has been useful for isolation, and MALDI-TOF MS can provide an accurate identification [11, 37]. An exhaustive species distinction using culture-based methods is difficult [11, 37, 38], and non culture-based methods, such as whole genome sequencing and microbiome analysis, will provide more detailed information on bacteria in polymicrobial cultures.

## Conclusion

Our findings suggest that *Streptococcus anginosus* group, *F. necrophorum*, *Prevotella* spp., and *S. pyogenes* are significant pathogens in the development of PPA. Additional, yet unspecified, anaerobes are also likely to play a role as are other less prevalent bacteria. Based on our findings, we recommend that the antibiotic regime for PPA patients include the coverage of the described likely pathogens, e.g., benzylpenicillin (to cover streptococci and *F. necrophorum*) and metronidazole (to cover *Prevotella* spp., *F. necrophorum*, and other anaerobes). We suggest utilization of a combination of highly focused culture-based and non-culture-based methods to comprehensively unveil the microbial pathogenesis of PPA.

## Data Availability

Anonymized data can be obtained from the corresponding author upon request.
